# Pneumonia, Treated with Quinia, with Illustrative Cases

**Published:** 1858-11

**Authors:** 


*Pneumonia, treated with Quinia, with Illustrative Cases.* By O. C. GIBBS, Frewsburg, Chautauque Co., N. Y.

In almost every disease it is not difficult to find good authority for divers to and even conflicting plans of treatment. It is certainly more respectful the opinions of others, if not more just, to suppose that each of such authorities may be right in their observations. It is more than probable that the varying causatives of disease, remote and immediate, develop varying grades of the same disease, or of the same pathological lesions. Climate, seasons, isothermal relations, epidemic and endemic influences, all exert varying influences upon diseased action, which, in the treatment of disease, it is all-important to heed. He who gives a cathartic at the outset of every case of fever, does so blindly and without cause; the same may be said of him who bleeds and gives antimony in every case of pneumonia, and the latter, in some seasons and localities, will certainly have no reason to boast of his success..

The above remarks were suggested on reviewing the cases of pneumonia that came under my observation for treatment during last winter and spring. From the first of November to the first of May last, I treated about thirty cases of pneumonia, none of which terminated fatally, and, with the exception of two, all were discharged cured in less than ten days from the date of first prescription. The first of these exceptional cases, was a gentleman, aged eighty-six years; the other that of a child of six years, with a congenital malformation of the heart, in which lung inflammation was developed in the course of a severe hooping cough. None of the thirty cases were bled, except two, which were seen in consultation, which bleeding, in both cases, was performed prior to consultation; a few only had antimony, by my order, and then in small doses, and during the first and second days; all had sulphate of quinine, and, in every instance, with the effect of lessening the frequency of the pulse, promoting perspiration, and loosening the cough.

Professor Austin Flint, of Buffalo, has, for some time, been treating pneumonia with stimulants; but it is due to myself to say that my prescriptions were made more in reference to present conditions than to precedent. I can better illustrate my plan of treatment and its results by cases than otherwise.

Case I. November 8, 1857, I was called to see Mrs. W——, aged about thirty-five years, of nervous temperament and feeble constitution, in consultation with Dr. Hollister. She had been under treatment about a week; she had had a calomel cathartic, which was followed by antimony, in such doses as the stomach would bear, and alterative doses of mercurials. At the time of my visit, she had a pulse 120 per minute, and easily compressible; her cheeks were flushed to nearly a purple color, but alternated with a deathly palor; cough was dry and expectoration difficult. It is needless to detail the symptoms, general and physical, suffice it to say, that the case was evidently one of pneumonia involving the left lung.

I advised powders, containing each two grains of sulphate of quinia and five of Dover's powders, to be given every four hours, and teaspoonful doses of a mixture, composed of equal parts of syrup of ipecacuanha and paragoric, to be given every four hours, commencing two hours after the administration of the first powder, A blister was ordered over the affected side. Dr. Hollister objected to the quinine, but, as I urged it, he gave way on my agreeing to assume the responsibility of the issue. At the urgent request of friends, I agreed to see the case on the second succeeding day. At that visit I found the skin moist, the cough loose and expectoration easy, tongue beginning to clean, the pulse reduced to 80, the pain in the side much mitigated, and the patient in every respect improved. The quinia and Dover's powders were ordered to be continued, and a decoction of senega with iodide of potassium was ordered instead of the syrup of ipecacuanha and paragoric. The patient recovered rapidly, and was discharged by the attending physician six days after this date.

Case II. December 11th, 1857, I was called to see Mr. M——, aged forty-five years, in consultation with Dr. Hollister of Corridon. On examination, I found the patient had pneumonia, involving nearly the whole of the right lung. He had been sick about eight days; he had been bled, had a calomel cathartic, and antimony had been pushed to the fullest extent the system would tolerate. The pulse was 130 per minute and very feeble; the tongue was swollen so as to retain the impressions of the teeth, and was covered with a smooth white fur overlaying its borders. Aphthæ occurred about the mouth and fauces, inclining the patient to the opinion that he had been injured by the administration of calomel. The cough was dry and grated harshly upon the ear. In consultation, the attending physician said that he had given antimony liberally, but he could not subdue the harsh cough, and produce expectoration. I thought he had given altogether too much, for, to me, it seemed as though the patient was prostrated nearly to the point where secretions are arrested. I ordered powders, containing three grains of quinine and six of Dover's powders, one to be given every four hours; also decoction of the senega and brandy sling, every four hours, with the addition of a few drops of laudanum, if the Dover's powders should not be sufficient to quiet all pain. A blister was also ordered to the affected side, but not to be applied until the patient was fairly under the influence of the opiate. At the earnest request the of attending physician and patient's friends, I saw the case on the second succeeding day. At this visit, I found the pain about the chest nearly gone, the cough was loose and expectoration easy, the skin was moist, the tongue had fairly begun to clean, and the pulse was reduced to 80: in a word, the patient was every way better, and freely expressed his satisfaction with the result of treatment and the prospect of recovery. I advised a continuance of the quinine and Dover's powders, and added the iodide of potassium to the senega—the brandy was continued. I saw no more of the patient, but was informed that his recovery was speedy and satisfactory; in fact, his improvement was so decided that he would allow his attending physician to make no change in my last prescription.

Case III. May 15th, 1858, I was called to see Mr. S——, aged about twenty years, in consultation with Dr. Smith, of Kennedyville. The patient had been ill about a week, of pluro-pneumonia; had been bled freely, been blistered, had a calomel cathartic, succeeded by alterative doses of mercury, and from the first, had antimony liberally.

The pulse was 130, skin dry, tongue clean, smooth, dry, cracked, and bleeding, and the teeth was covered with dark sordes. I advised quinine and Dover's powders in combination, three grains of the former and five of the latter, to be administered every four hours, two grains of iodide of potassium indecoction of senega, every eight hours, and fifteen drops of balsam of copavia, also every eight hours. Dr. Smith objected to the quinine, as an unheard of innovation, and altogether hazardous in the treatment of a local inflammation. I urged the point, and predicted death without it or an efficient substitute. He consented to adopt my suggestion, with the privilege of discontinuing should the pulse increase in frequency. At the request of attending physician and patient's friends, I engaged to see the patient again on the second succeeding day. At that visit the pulse was 85, tongue and skin moist, and the patient in every way improved. The treatment was ordered continued. I saw no more of the case, but was informed that the recovery was speedy and perfect.

In the cases above, it may be said that quinine treatment was brought to bear only after the inflammation had been subdued by bleeding, antimony, and mercurials, and that the treatment thus instituted, at the end of the first week, would have been decidedly injudicious at the outset. My experience inclines me to the opinion, that in the cases given above, the early treatment only increased the danger to life, and protracted the recovery. in my first cases, quinine and opium was only brought to bear upon the second or third day; but subsequently this treatment was only preceded by a cathartic. In every case, the frequency of the pulse became less immediately after commencing with the quinine. One or two cases will illustrate the treatment and its results.

Case IV. March 2d, 1858, was called to see a lad about twelve years old. He had had a chill the day before, had now flushed cheeks, headache, pain in the right side, cough, &c. After a careful examination, the case was diagnosed as one of pneumonia, involving the lower half of the right lung. A cathartic of hydrargyrum cum creta and rhubarb was ordered, to be followed by epsom salts sufficient to secure a free catharsis. This was to be followed by three grains of Dover's powders every four hours, and small doses (about one-twelfth of a grain) of antimony, in a solution of citrate of potash, every four hours.

On the next day, the symptoms were much the same. The sputa was decidedly rust-colored, the pain in head and side was less, the pulse was softer, but retained its former frequency. I now ordered a blister to the affected side, added one and one-half grains of quinine to the Dover's powders, and, as an expectorant, instead of the antimony, I substituted a mixture composed of syrups of senega, ipecac., and liquorice.

On the next day, the third of treatment, I found the skin moist, the cough loose, the pulse reduced to 80, and the tongue beginning to clean. The treatment of yesterday was ordered continued. On the fourth day, the patient doing well, the syrup of senega and ipecac was discontinued, and decoction of senega with iodide of potassium substituted. On the sixth day the patient was discharged, on being advised to continue treatment for a few days longer.

I had intended to report other cases, but, on looking them over, I find them so exactly alike, as to treatment and results, as to make one but a repetition of the other. In several cases, the quinine and Dover's powders followed immediately after the cathartic, with the effect of reducing the pulse in the first twenty-four or thirty-six hours.

The only exceptions to the prompt and decided curative results, mentioned in the last case reported, were the two cases mentioned early in this paper; both of which, I doubt not, would have died under the treatment sometimes unwisely pursued in pneumonia.

In the cases reported, I have not entered minutely into the symptoms, general and physical, upon which diagnosis was based; I have presumed that my readers would concede to me the ability to make correct diagnosis; suffice it to say that the diagnosis was established from physical signs, and confirmed, except in quite young children, by the rust colored sputa.

I would not be understood as stating that this treatment is adapted to all seasons, nor to all localities; but would simply make this record of my experience, that others may derive such practical lessons as are legitimately deducible from the premises.

It is proper to observe here, that my cases, though occuring mostly in the in the valleys of the Allegheny and the Connewango rivers, could have but little or no connection with malarious influences, for an intermittent fever I have never known to originate in the present range of my practice.

---

*The Diseases of Quinine Makers.*—M. A. Chevallier, at the last sitting of the Academy of Sciences of Paris, communicated a paper on the diseases to which workmen employed in the manufacture of sulphate of quinine are subject. It appears from his statement that one of the disorders is a cutaneous affection severe enough to force them to suspend work for a fortnight, a month, or sometimes altogether. M. Chevallier further quotes M. Zimmer of Frankfort to testify to a particular kind of feverbark fever (das China Fieber) which effect workmen engaged in pounding bark. This has not yet been observed in France. It is described as being so painful that those who have once suffered throw up employment rather than risk a second attack. As for the cutaneous effection, it attacks not only workmen, but those about the place, and affects alike the sober and the intemperate. No remedy has as yet been discovered.—*Lancet.*

---

*Excessive Nausea during Pregnancy Relieved by Cauterizing the Cervix Uteri.* By Dr. PARKS.

Dr. Parks remarked, that he gave the following abstract in consequence of a somewhat similar case reported by Dr. Churchill, in the Dublin Quarterly for August last, and which was stated to be, so far as Dr. C. knew, the first instance in which the nausea of pregnancy had been treated by applications to the cervix uteri. Dr. Churchill's case, however, occurred in November or December; that now reported, in January, 1856, and was read at a meeting of the society for medical observation, before the publication of Dr. C.'s case.

Jan. 17th. 1856. Mrs. ——, aged about twenty-seven years, consulted Dr. P. for excessive nausea during pregnancy. She was rather a delicate woman, though active and energetic. At one time, since marriage, she had uterine symptoms, which were referred to inflammation of the uterus by the practitioner under whose care she was, and who treated the patient locally, with the aid of the speculum. The result was successful, so far at least as the symptoms were concerned. In other respects she had been well, till the occurrence of a miscarriage, which took place a year or more since, she says, from some accidental cause.

The patient had not menstruated for three months, and had begun to enlarge as if from pregnancy. She had also, for a considerable time, been troubled with nausea, which had become almost constant, and so troublesome as to lead her to seek advice in relation to it. On examination with the speculum, an abrasion was found—marked, but not severe—at the os tincæ, which was cauterized with nitrate of silver. Two days after, the patient stated that the nausea was greatly alleviated immediately after the operation, and on the day following had almost entirely ceased, now only existing to a slight extent. A mixture, containing lavender, gentian, and ginger, was prescribed. The patient went her full time comfortably, and had a safe delivery.

Dr. Storer mentioned two cases of this affection, which were relieved by the application to the cervix uteri of the saturated tincture of iodine. He had also tried the tincture of iodine internally in doses of three drops, and found it even more effectual than creosote.—*Ext. Bos. Soc. for Med. Imp. Virginia Med. Journal.*—From *St. Louis Med. and Surg. Jour.*

*Hydropneumothorax treated by Paracentesis Thoracis and Injections of Iodine.*—Three cases of this under the care of Prof. Trousseau are recorded *L'Union Médicale* (Oct. 31, and Nov. 10, 1857,) one of which proved fatal, and the other two resulted favorably. In the former, death did not ensue till six weeks after the operation; the disease for which it was performed having been complicated with phthisis, which was not positively diagnosed during life, it would not be just to impute the fatal results to the operation.

The most completely successful of the three cases was the second, of which the following is an outline: A man was admitted into the hospital, Nov. 12, 1856, six weeks after having suffered serious injury by being squeezed between two carts. He was still in great pain, the right side of the thorax presented a considerable elevation; there was dullness posteriorly as far as the spine of the scapula, and the respiratory murmur was inaudible at the base of the chest; in the infra-spinous fossa there was a blowing sound, ægophony, metallic tinkling, and hippocratic succussion caused a splashing which was audible at a small distance from the thorax. In the subclavicular space the percussion sound was abnormally clear. The abdominal organs were forced downwards, the liver projecting considerably below the false ribs. There was a frequent cough with expectoration, which was at times bloody and rusty, at others frothyand colorless; respiration was quick and painful, the pulse small, about 120. The patient's strength was good. M. Trousseau diagnosed hydropneumothorax, and on introducing a trocar between the seventh and eighth intercostal space, 5½ litres (nearly twelve pints) of an odorless purulent fluid were evacuated; and having entirely emptied the pleura, 258 grammes of an iodized solution were injected, which was partly removed after a few minutes, and the wound shut up. No inconvenient symptom followed; the wound healed up in the course of eight weeks, during which time it continued to discharge a purulent serosity. The chest by this time had collapsed on the right side; the vesicular murmur was everywhere restored, but feeble, and mixed with mucous rales in some points posteriorly; in front it was pure and audible, even on the level of the puncture. The patient left towards the end of January, and soon after was well enough to return to his employment as cartman.

The third case occurred in a man, aged thirty-five years, as the result of pleurisy of the left side. The operation was performed as in the last case, followed by iodized injections,* repeated no less than twenty-four times at different intervals. The patient did well; the left side of tho chest fell in, and the percussion sound of the part affected was restored almost to the normal condition; but five months after the operation the orifice made by the trocar still continued to discharge a small quantity of pus, and it was feared that there was tubercular deposit in the lung, to combat which the hypophosphite of lime was prescribed according to the formula of M. Churchill.

In the remarks that conclude the article, the propriety of performing the operation is dwelt upon, and the iodized injections are spoken of as the only remedy by which we may hope to obtain a cure of purulent effusions, and of hydropneumothorax even, without serious danger.—*B. and F. Med.-Chirurg. Rev.* Jan, 1858.

---

* Fifty grammes of tincture of iodine, five grammes of iodide of potassium, with one hundred to one hundred and twenty grammes of water.

*After Treatment of Surgical Operations.*—Dr. Broadbent, in a paper read before the Liverpool Medical Society, session 1855-56, referred to the object sought—that of union by the first intention. He believed one most common obstacle, to this union to be the occurrence of hemorrhage, one or two hours after the operation; not to such an extent as to require the removal of the dressings, but sufficiently to form a coagulum of such a size as to seriously interfere with the union of the wound—acting, in fact, as a foreign body. The cause of this appeared to be, that in amputations, &c., when the surfaces were brought together immediately after the completion of the operation, the vessels were tied while the patient was still suffering from the shock of the operation, or it might be, was somewhat depressed by the after effect of chloroform, whilst the more minute vessels were prevented from oozing, by their exposure to the air, and that, when reaction took place, the hemorrhage came on. The author thought that those cases in which this occurred to such an extent as to necessitate the reopening of the wound, usually terminate more favorably than others. He therefore advocated the plan of postponing the dressing until all oozing had ceased, and the cut surfaces had glazed over. The unnecessary removal of dressings, he believed to be another frequent cause of non-union. He thought that the sutures having been removed, the bandages, &c., should remain untouched till the third or fourth day, and should then be carefully cut off. The inability of the patient to maintain the required position, acted in the same way, and to obviate this, it was suggested that the patient should, before the operation, be habituated to the position in which he would have to lie after it. In the maintenance of the position of the parts, by means of pressure, the author believed that small air-cushions might be advantageously used, instead of pads of lint.—*Liverpool Medico-Chirurgical Journal*, July, 1857.

---

*Belladonna in Juvenile Incontinence of Urine.*—The use of belladonna against incontinence of urine in children, as strongly recommended about a year ago by Mr. Brooke, of the Westminster Hospital, has, we believe, well borne the test of the trials which his laudation of it induced. Several surgeons have, we know, formed most favorable opinions of its efficiency. A case under Mr. Hutchinson's care, at the Metropolitan Free Hospital about three months ago, afforded very conclusive evidence of its power. The patient was a boy of ten, who had from infancy been exceedingly troubled by inability to retain his water. Nightly incontinence was a matter of rule, and very often the urine would escape during the daytime also. Nux vomica, sesquichloride of iron, etc., had been fairly tried, and without benefit. At first the belladonna seemed to do no good, but being pushed until symptoms of poisoning were apparent, it finally effected a complete cure. The bladder appeared to have wholly lost its morbid irritability, and during six weeks that the boy remained under observation, his mother stated that no single instance of incontinence had occurred. The remedy was given in solution in water, and without any adjuvant whatever. Belladonna is one of our remedies which certainly deserves a more thorough clinical investigation of its powers than it has yet received.—*Med. Times and Gazette*, July 31, 1855, from *Med. News and Library.*

